# Bali Belly: *Salmonella senftenberg* Found in an Infected Ovarian Endometrioma

**DOI:** 10.1155/2020/3581091

**Published:** 2020-08-24

**Authors:** P. D. M. Pathiraja, Junaid Rafi, Emily Woolnough, Anna Clare

**Affiliations:** ^1^Ministry of Health, Sri Lanka; ^2^St John of God Midland Public and Private Hospitals, Australia

## Abstract

Salmonella is an extremely rare cause of an infected endometrioma. We present a case of a 30-year-old immunocompetent woman presenting with fevers and abdominal pain, on a background of prior endometriosis. Initial antibiotic treatment for pelvic inflammatory disease failed, and the patient progressed to septic shock requiring surgical evacuation of an infected ovarian endometrioma. Microbiological samples from stool, ovary, and peritoneal fluid revealed infection with *Salmonella senftenberg*. The likely diagnosis was Salmonella enterocolitis with bacterial translocation to an ovarian endometrioma.

## 1. Case Report

A 30-year-old nulliparous woman presented to the emergency department with sudden onset of generalized lower abdominal pain, mild fever, and vomiting for one-day duration on a background of three days of diarrhea. She had associated generalized aches and headache. She had a past medical history of endometriosis. She was otherwise generally fit and well, worked in administration on a remote mine site, and had recently returned from a holiday in Bali. On assessment, her temperature was 37.9°C, and she was hemodynamically stable. An abdominal examination revealed tenderness over the suprapubic area. A vaginal assessment showed a normal sized uterus and severe right-sided adnexal tenderness with no abnormal discharge.

**Figure 1:**
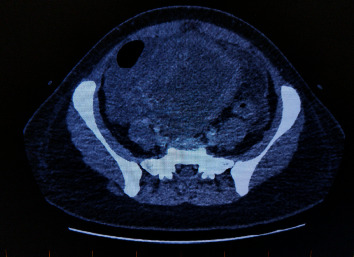
CT report of the abdomen and pelvis. Large pelvic mass which may be ovarian in nature. The fluid content of this cystic lesion is 24 HU in density. It has a thick irregular wall. There is generalized ascites and peritoneal induration.

Her inflammatory markers were raised (white cell count 23 × 10^9^/L, neutrophil 21 × 10^9^/L, C-reactive protein 233 mg/L). A computed tomography of the abdomen (CT abdomen) and pelvis (Figure 1) showed a possible tubo-ovarian abscess secondary to an ovarian cyst or endometrioma 111 × 118 × 95 mm in size. Her liver and renal function was normal. Endocervical swabs for chlamydia and gonorrhea polymerase chain reaction (PCR) were negative, and a high vaginal swab was sent for microbiology showed normal flora. Of note, a stool PCR was positive for Salmonella species; the significance of this was uncertain. The initial diagnosis was pelvic inflammatory disease, and she was treated with intravenous ceftriaxone, metronidazole, and azithromycin as per local guidelines. After three days of antibiotics, she was well and discharged with oral azithromycin to complete treatment for possible mild Salmonella enteritis. Follow-up in the gynecology clinic was planned to discuss the ongoing management of endometriosis.

**Figure 2:**
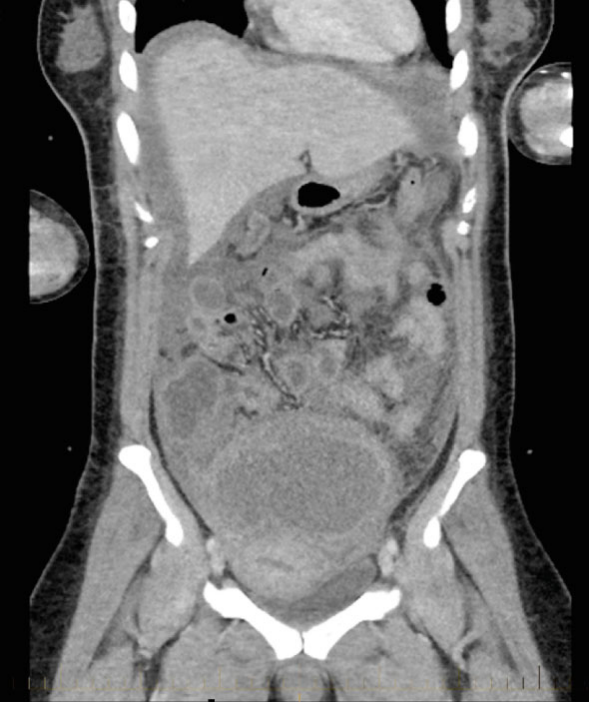
CT report of the abdomen and pelvis. There is a large cystic mass arising off the pelvis, which is complex in appearance. The wall is thick and irregular; this lesion has axial/transverse dimensions of 12.6 × 13.6 cm, with a sag/oblique long axis of 15.4 cm.

However, four days later, she presented with an acute surgical abdomen and septic shock requiring inotropic support. The CT of the abdomen and pelvis revealed a large pelvic mass (120 × 130 × 150 mm in size) with copious free fluid in the pelvis and edematous bowel (Figure 2). She had an emergency laparotomy and drained 1500 ml of endometriotic fluid from the peritoneal cavity. There was inflammatory exudate present over the bowel wall, and when the endometrioma was laid open, it was noted to be purulent and malodourous. A peritoneal fluid was sent for culture and grew *Salmonella senftenberg*. A cystectomy of the endometrioma was performed, and histology of the cyst wall was compatible with endometriosis. Infectious disease consultation was sought. She had nine days of intravenous antibiotics and was discharged on a three-week course of high dose oral ciprofloxacin, to which the organism was susceptible. The patient had recovered well on review in clinic.

## 2. Discussion

The Salmonellae are a family of gram-negative motile bacilli, of which two serotypes cause severe enteric fever—S. typhi and S. paratyphi. The remainder is collectively known as the nontyphoidal Salmonellae, and this includes Salmonella senftenberg. Enteric fever due to typhoidal Salmonella is a major global health problem, which causes 200000 deaths annually and further significant morbidity [1]. Patients with nontyphoidal Salmonella typically present with self-limiting gastroenteritis. Less than 5% of patients with Salmonella gastroenteritis develop bacteremia and distant spread.

Salmonella is transmitted via the fecal-oral route from contaminated food, most commonly poultry, milk, and eggs [2]. Direct animal contact and person-to-person transmission are rare. The incubation period for salmonellosis is 12–72 hours. The typical initial symptoms are fever, diarrhea, abdominal pain, and generalized myalgia.

Salmonella can breach the barrier of the intestinal cell wall through the process of phagocytosis and then enter macrophages through micropinocytosis. The microorganism then disseminates through the body via hematogenous spread and can metastasize to distant sites. These complicated disseminated cases require prolonged antimicrobial therapy.

The most common sites of secondary involvement are the bone, lung, heart, spleen, and kidney. Ovarian involvement is rare. In this case, a spread of infection to the ovaries could have been due to direct contact with inflamed bowel, through hematogenous spread or via ascending infection. Those with immunodeficiency conditions are at higher risk of aggressive and disseminated disease [3–5]. There have been previous case reports of Salmonella presenting during pregnancy with ovarian infection and mimicking a gynecological malignancy [6, 7].

A literature review revealed nearly thirty cases of ovarian involvement reported over the past five decades. *Salmonella enteritidis* and *Salmonella typhi* are the subtypes most frequently isolated from the ovary. *Salmonella senftenberg* infection in an ovary is unreported. In previous case reports, a wide range of antibiotics have been used for treatment, including cephalosporins, fluoroquinolones, and macrolides. Increasing rates of Salmonella resistance to fluoroquinolones are reported, particularly in Asian regions [8].

## 3. Conclusion

This case likely represents Salmonella enterocolitis with translocation to a preexisting endometrioma, leading to cyst rupture and septic shock. The case alerts the gynecologist that salmonella infection can manifest in multiple organ systems and highlights the importance of a broad differential diagnosis in a patient with a presentation of fevers and abdominal pain.
